# Supplementary material for: Nonlinear optical vector processing using linear silicon photonic circuits for 50 Gb/s memory and string similarity functions
Source: Nat Commun. 2025 Dec 17;16:11416. doi: 10.1038/s41467-025-66286-7 (PMC12749030; doi:10.1038/s41467-025-66286-7)
Supplement: Supplementary file 1 — Supplementary Information [file 41467_2025_66286_MOESM1_ESM.pdf]

Supplementary material for

Nonlinear Optical Vector Processing using Linear Silicon Photonic Circuits for 50 Gb/s Memory and String Similarity functions

T. Moschos*[1,2], C. Pappas[1,2], S. Kovaios[1,2], I. Roumpos[1,2], A. Prapas[1,2], A. Tsakyridis[1,2], M. Moralis-Pegios[1,2], C. Vagionas[1,2], Y. London[3], B. Tossoun[4], T. Van Vaerenbergh[3] and N. Pleros[1,2]

[1]*Department of Informatics, Aristotle University of Thessaloniki, 54124, Thessaloniki, Greece*

[2]*Center for Interdisciplinary Research and Innovation, 57001, Thessaloniki, Greece*

[3]*Hewlett Packard Labs, 820 N McCarthy Blvd, Milpitas, CA 95035, USA*

[4]*Hewlett Packard Labs, Santa Barbara, CA 93106, USA*

*moschost@csd.auth.gr

1. Principle of operation for single and WDM optical comparison cells

The proposed layouts of the single and multi-wavelength comparison cell, along with their basic principle of operation are demonstrated in Supplementary Figure 1. Supplementary Figure 1 (a) presents the schematic of the single comparison unit, which consists of a pair of thermo-optic (TO) intensity modulator (IM)-based nodes, followed by TO phase shifters (PS). The optical search vector encoding unit (SVEU) is responsible for generating the search bit values as a complementary non-return-to zero (NRZ) pattern (Bit and $\overline{\text{Bit}}$). The cell is configured by adjusting the biasing of the IMs, enabling it to encode three distinct target symbol information states: i) “0”, ii) “1”, and iii) the ternary value, or ‘don’t care’ state “X”, which is widely utilized in ternary content addressable memories (TCAMs). Supplementary Figure 1(b) illustrates the configuration of the WDM comparison cell, which encodes different target symbol values by assigning each value to a specific wavelength. In both cases, the final output of the column reflects the result of the logical comparison operations based on the cell's configured static information, indicating either a logical “match” or the degree of “mismatch” in terms of optical power. Specifically, a zero-power pulse amplitude represents a logical “match”, signifying identical compared

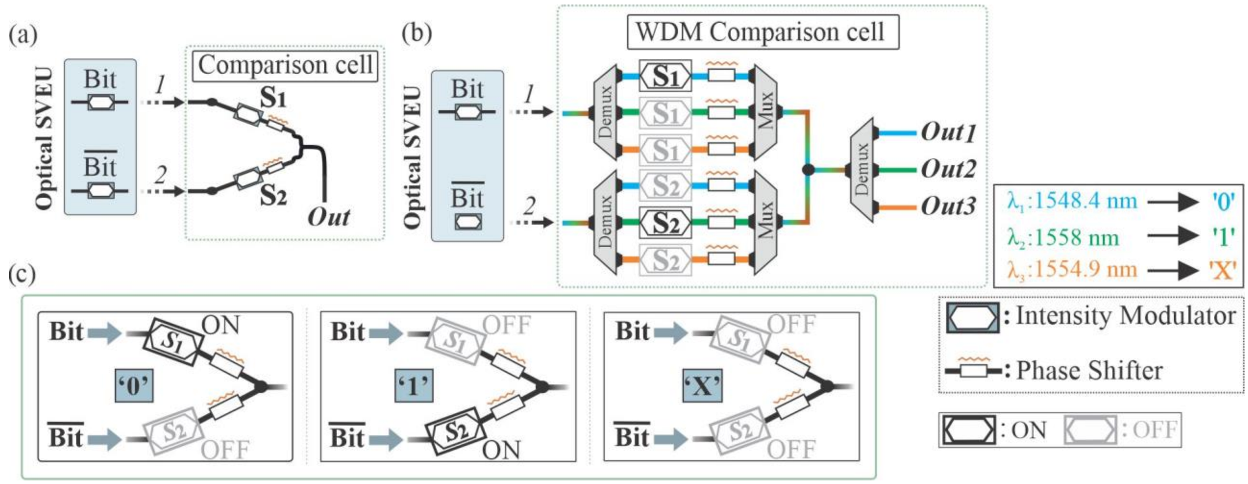

Supplementary Fig. 1. (a) Optical single comparison cell and (b) WDM comparison cell configuration. (c) Logical symbol content assignment for the logical symbol values of “0”, “1” and “X”.

Supplementary Table 1: Truth table of Binary and Ternary symbol encoding of the comparison cells

Search Bit	Search Bit Optical Encoding		Stored Symbol	Stored Value Optical Encoding		Logical Decision	Comparison cell Output
	Bit	$\overline{\text{Bit}}$		S_1	S_2		
0	0	1	0	ON	OFF	Match	0
1	1	0		ON	OFF	Mismatch	1
0	0	1	1	OFF	ON	Mismatch	1
1	1	0		OFF	ON	Match	0
0	0	1	X	OFF	OFF	Match	0
1	1	0		OFF	OFF	Match	0

values, whereas a non-zero power indicates the degree of "mismatch", revealing the power-level difference between the incoming search value and the target symbol. Supplementary Figure 1(c) shows the logical cell assignment for the respective symbol values. In the single-cell unit, the incoming optical signals for the search bit and its complement i.e. Bit and $\overline{\text{Bit}}$, propagate through the two optical branches of the cell, each containing an electro-absorption modulator (EAM), denoted as S_1 and S_2. The TO phase shifters (PSs) following the EAM modules ensure proper phase matching by facilitating the constructive interference of the optical branches at the common output. The EAMs are configured to either the OFF or ON state by controlling their bias to activate or deactivate the absorption condition. In the single wavelength case, by applying a reverse voltage of 4V to the respective EAMs the output optical signal is suppressed, ensuring the signal does not exit the EAM. Conversely, if no voltage is applied, the propagated signal remains unsuppressed and is visible at the module's output. Subsequently, the two coherent optical beams are being coupled together via a 2:1 combiner, with the resulting output signal carrying the dot-product between the target symbol and the search bit value. In the case of the WDM cell, the search information is distributed to all respective S_1 and S_2 EAM pairs for parallel comparison. The cell symbol assignments follow the same configuration logic as the single wavelength comparison cell, with the key difference being that the EAMs are reverse-biased with a voltage of 3V. The multiplexer modules forward the results of the three different comparisons to the common output of the cell, where each specific case can be monitored by isolating the corresponding wavelength using a filtering element (demultiplexer or optical filter). As shown in Supplementary Fig. 1 (c), to configure the logical "0" symbol, the two EAMs have to be set as [S_1=ON, S_2=OFF]. Following the same rationale, the logical "1" is represented by [S_1=OFF, S_2=ON] configuration, while the logical "X" state corresponds to the [S_1=OFF, S_2=OFF] configuration.

Supplementary Table 1 provides a detailed truth table of the proposed layout, summarizing all possible combinations of the incoming search and target symbol values, the respective encoding, and the final cell comparison outputs. In this table, a "0" value represents a zero-level output power, indicating a logical "match" while a "1" value represents a non-zero output power, indicating a logical "mismatch". This table represents all combinations derived from a 1-bit/symbol comparison and can be extended by using larger optical SVEUs, with each pair of complementary modulated values providing additional search capacity. The number of target vectors that can be encoded in this architecture can be increased either physically, by incorporating additional columns, as in the case of the 4×4 Xbar architecture, or by utilizing the wavelength degree of freedom provided by WDM.

2. Multiplexer and Demultiplexer modules of the WDM Comparison cell

The multiplexer and demultiplexer components that are used in the implementation of the WDM-enabled HD circuit, comprise 2nd order micro ring resonators (μRRs) [1], operating as filtering elements. Supplementary Figure 2(a) presents the circuit layout of the proposed WDM-based interferometric cell, including the respective multiplexer and demultiplexer

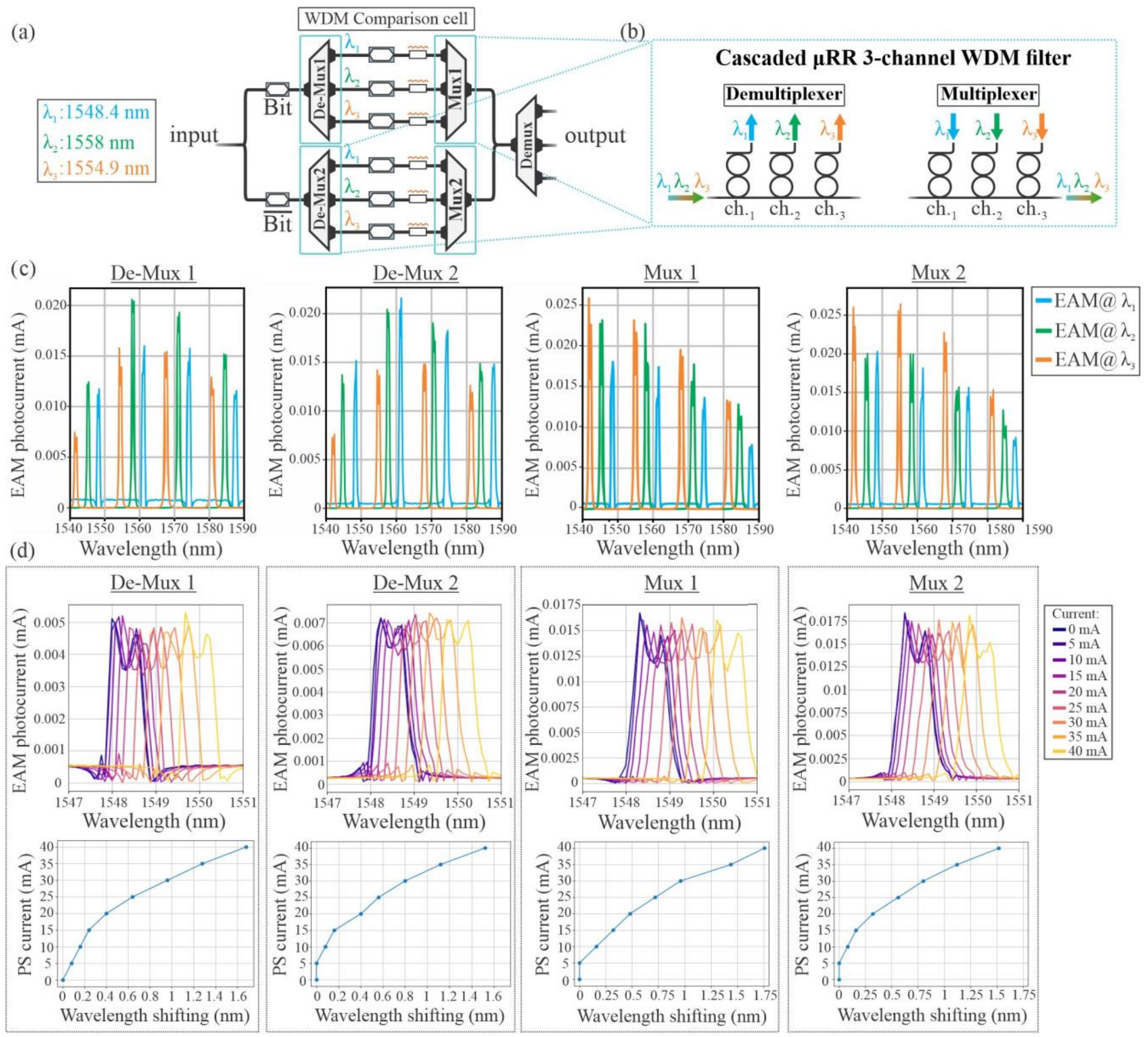

Supplementary Fig. 2. (a) Circuit layout of the WDM comparison cell, (b) Layout of cascaded 3-channel μRR filters as Multiplexer and Demultiplexer modules, (c) EAM photocurrent (mA) in response to wavelength, for all the Mux/Demux modules, (d) Wavelength shifting response of the respective ring structures for specific applied current values.

(Mux/Demux) elements. Supplementary Figure 2 (b) shows the layout of the three channel μRR stages of the filter structures. In the demultiplexer case, the WDM stream consisting of the wavelengths λ_1=1548.4 nm, λ_2=1558 nm and λ_3=1554.9 nm, enters through a common input into the structure, with each of the three wavelengths being filtered at the output of every double ring channel. On the other hand, in the multiplexer scenario, the separated signals are inserted into each double ring channel and are then multiplexed in a common bus output. Towards investigating the spectral response of the demux/mux, we have exploited the electro-absorption effect of EAMs to capture the photocurrent generated by an incident optical beam. Initially, an optical signal, generated by a tunable laser source (TLS), was injected through the input port, to evaluate the De-mux$_1$ and De-mux$_2$ spectral behaviour. Once the optical signals reach the respective EAM modules, after the demultiplexing stage, the photocurrent produced by the EAMs in response to different wavelength values is measured, with the corresponding diagrams shown in Supplementary Figure 2 (c). To test the spectral response of Mux$_1$ and Mux$_2$, we injected an optical signal from the output port (respective filtering port for every wavelength case) while again monitoring the EAMs' photocurrent. The diagrams indicate a misalignment in the wavelength tuning between the Mux/Demux modules, requiring adjustments to fully align the structure grid. By tuning the modules of De-mux$_1$, De-mux$_2$ and Mux$_1$ (De-mux$_1$ → 18 mA current applied, De-mux$_2$ → 15 mA current applied and Mux$_2$ → 12 mA current applied), through dedicated thermo-optic PS elements, while keeping Mux$_2$ as a reference, complete alignment can be achieved.

Supplementary Figure 2 (d) presents the wavelength shifting response of every Mux and De-mux module along with its TO PS current requirements. One of the EAMs in each branch of the interferometer is indicatively selected as a reference element for its photocurrent response for each μRR filter. In every Mux and Demux, current values in the range of [0-40 mA] have been applied, revealing a maximum wavelength shift of: 1.7 nm for the De-mux$_1$, 1.5 nm for the De-mux$_2$, 1.75 nm for the Mux$_1$ and 1.5 nm for the Mux$_2$.

3. Noise analysis of single wavelength and WDM-enabled HD processors

The architectures under study are evaluated both as CAM operators that validates the matching conditions when the HD=0, as well as HD calculation units producing multi-level signals that correspond to the HD between the two optical vectors. The evaluation of the single wavelength and WDM Xbar approaches has been performed for different crossbar (Xbar) scales ranging from [4,128], as well as for the different operational rates of 20 and 50 GHz. The performance is validated through the calculation of match error rate (MER) when CAM operation is targeted and the symbol error rate (SER) when HD calculation is performed. In the current analysis three different noise sources are considered, specifically being: (i) the relative intensity noise (σ_{RIN}) that stems from the noise contribution of the laser source, (ii) the shot noise (σ_{SHOT}), produced by the current random fluctuations of electrons that occur into the photodiode (PD) module and (iii) the thermal noise ($\sigma_{THERMAL}$) which is mainly produced by the transimpedance amplifier (TIA), at the receiver site[2]. Supplementary Figure 3 shows a layout approach that includes all the noise components and their contributing noise factors. The noise components are approached and modeled as zero-mean additive white Gaussian noise sources, based on the central limit theorem[3].

Moreover, different signal power levels have been taken into consideration for the calculation of noise, depending on the different match/mismatch levels of the Hamming distance/CAM comparison operations. This approach allows for more accurate estimations of the noise profiles for the multi-level comparison outputs, since the shot and RIN noise closely depend on the received power of the signal, meaning that different noise characteristics will be assigned to different signal levels, following accurately the principle of the Hamming distance calculator. In the current implementation, the signal levels correspond to comparisons of different symbol lengths supported by different crossbar scales [4,128].

For the calculation of the discrete signal comparison levels, the following equation is followed:

$$P_{\text{Signal level}}\ (\text{mW}) = P_{\text{laser}}\ (\text{mW}) - (IL_{\text{Crossbar}}\ (\text{dB}) + IL_{\text{signal level}}\ (\text{dB}) + 3\ (\text{dB})) \qquad (1),$$

where P_{laser} is the input laser power that is inserted into the crossbar table, for every different laser power under study in a range of 40 dB (-10 to 30 dB). The Xbar losses are calculated using the mathematical equation presented in[4], while the signal level losses correspond to the differences in signal power levels. These losses assume an equal power distribution, where the highest level of "mismatch" corresponds to the highest power value, and the "match" state results in a near-zero power output. Finally, the additional 3 dB losses are attributed to the driving conditions of the search encoding EAMs, as half of them will be configured to "0" values at any given time instance, based on the Bit and its complementary $\overline{\text{Bit}}$ value.

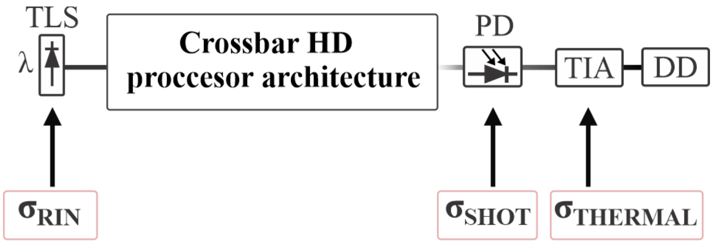

Supplementary Fig. 3. Layout of the Crossbar Hamming Distance processor architecture's noise sources. The electro-optic components that contribute to the overall noise profile are: the laser diode (TLS), the photodiode module (PD) and the transimpedance amplifier (TIA).

After calculating all the respective signal levels, the calculated powers are converted to a receiver photo current ($I_{received}$), based on the responsivity (R) of the photodiode (PD), which in our case is considered to be 0.8 A/W, based on the equation:

$$I_{received} = P_{Signal\ level}\ (\mathrm{mW}) \cdot R \quad (2),$$

Based on the previous calculations, the noise parameters can be calculated through the following Supplementary equations:

$$\sigma_{RIN} = I_{received} \cdot \sqrt{\mathrm{RIN} \cdot \mathrm{B}}$$

$$\sigma_{SHOT} = \sqrt{2 \cdot \mathrm{e} \cdot \mathrm{I_{received}} \cdot \mathrm{B}}$$

$$\sigma_{THERMAL} = \text{spectral density} \cdot \sqrt{\mathrm{B}} \quad (3),$$

where B stands for the operational bandwidth, e for the electron charge parameter in the photodiode, RIN for the relative intensity noise density of the laser source, while the spectral density refers to the TIA module. Based on state-of-the-art high-bandwidth electro-optic components we considered the following values of: RIN = -150 dB/Hz, e = 1.602 e^{-19} and spectral density = 15.084 pA/$\sqrt{\mathrm{Hz}}$. The total noise value for every signal level is then calculated as:

$$\sigma_{total\ noise} = \sqrt{\sigma^2_{\mathrm{RIN}} + \sigma^2_{\mathrm{SHOT}} + \sigma^2_{\mathrm{THERMAL}}} \quad (4)$$

The comparison performance relies on XOR logical operations, which are summed at each comparison output. Specifically, the XOR operation is performed between the search and stored words, each consisting of n symbols, whose values follow a uniform distribution. The bit-by-bit comparison yields a '1' when the values differ and a '0' when they are equal. For every compared symbol stream, the count of positions that have a difference, is described as a Binomial distribution[5] when assuming a uniform distribution of the binary values of the two compared binary strings. This is expressed as Binomial (n, p), where n is the respective number of symbols and p is the probability of success, with an equal probability of 0.5 (p=0.5) for the bits 0 and 1. The binomial distribution can then be expressed as:

$$\text{P(Hamming distance = k)} = \binom{\mathrm{n}}{\mathrm{k}} \cdot p^k \cdot (1-p)^{n-k},$$

with the binomial coefficient, representing the number of ways to choose k successes out of n trials, with p^k being the probability of each symbol differing and $(1-\mathrm{p})^{\mathrm{n-k}}$ the probability of n-k failures. Based on the different length of the sequences that can be supported by every crossbar scale, a different probability of occurrence characterizes every different signal level at the output of the HD matching operator. For an example case of a 4-symbol word, the following probability responses are shown:

- For an n value equal to 4 (n = 4), the binomial distribution is configured as P(Hamming distance = k) = $\binom{4}{k} \cdot 0.5^{\mathrm{k}} \cdot (0.5)^{4-\mathrm{k}}$ ⇒ P(Hamming distance = k) = $\binom{4}{k} \cdot 0.5^4$, with the following cases characterizing the probability of every Hamming distance occurrence:
- P(k=0) = $\binom{4}{0} \cdot 0.5^4$ ⇒ P(k=0) = 0.0625, refers to the case of a complete match, with a Hamming distance that equals to "0" (HD=0)
- P(k=1) = $\binom{4}{1} \cdot 0.5^4$ ⇒ P(k=1) = 0.25, refers to the case where 1 compared symbol differs, with a Hamming distance that equals to "1" (HD=1)
- P(k=2) = $\binom{4}{2} \cdot 0.5^4$ ⇒ P(k=2) = 0.375, refers to the case where 2 compared symbols differ, offering a Hamming distance that equals to "2" (HD=2)
- P(k=3) = $\binom{4}{3} \cdot 0.5^4$ ⇒ P(k=3) = 0.25, refers to the case where 3 compared symbols differ, meaning that the Hamming distance will equal to "3" (HD=3)

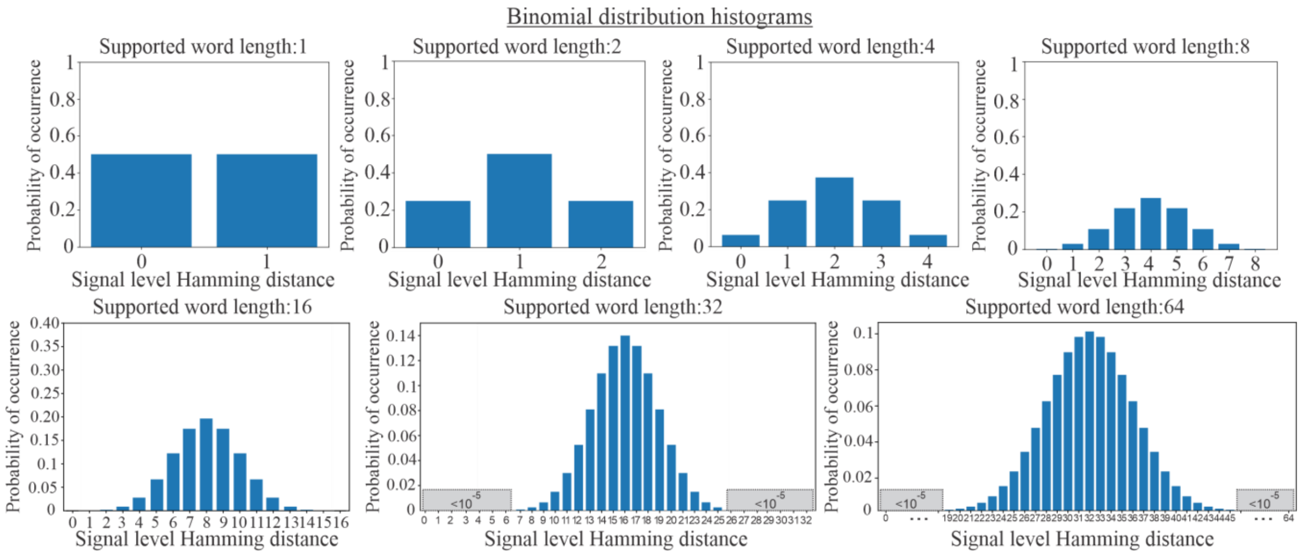

Supplementary Fig. 4. Hamming distance Binomial distribution histograms of signal level's/symbol sequence probability of occurrence at the comparison output of the multi-word comparison operator.

- P(k=4) = $\binom{4}{4} \cdot 0.5^4 \Rightarrow$ P(k=4) = 0.0625, refers to the case of a complete mis-match, with the highest value of Hamming distance (HD=4)

Following the same principle for all the different crossbar cases and their supported words, Supplementary Figure 4 presents all the different Binomial distribution histograms.

Following the calculation method for the signal level probabilities and the noise parameters that correspond to each power level, the error rates calculation method is expalined.

The calculation of the MER is based in the following equation:

$$\mathrm{MER} = \mathrm{P}(\mathrm{x} \,/\, 0) + \mathrm{P}(0 \,/\, \mathrm{x}) \qquad (5),$$

where P(x / 0) expresses the bound false positive probability of detecting an x symbol instead of the actual symbol "0", while the P(0 / x) is the false negative probability of detecting a "0", when expecting another x symbol. Every probability is calculated using the complementary error function (erfc), representing the tail probability of a Gaussian distribution. The extracted probabilities of occurrence for every symbol are used to weight the partial bound probabilities, with *p(0)* used for the signal's 0-level, while for all the other cases the respective probabilities are summed for the multiple i levels (from 1 to M-1), weighting each bound probability by the respective *p(i)* probability of occurrence.

Additionally, in order to optimize the MER measurements, we calculate the optimum threshold value, noted as *t*, between the zero-level and all the other signal levels, using the derivative of the MER with respect to the threshold, as follows:

$$\frac{\partial(\mathrm{MER})}{\partial(\mathrm{t})} = \frac{\partial(\mathrm{P}(\mathrm{x}/0))}{\partial(\mathrm{t})} + \frac{\partial(\mathrm{P}(0/\mathrm{x}))}{\partial(\mathrm{t})} \qquad (6),$$

where the $\frac{\partial(\mathrm{P}(\mathrm{x}/0))}{\partial(t)}$ is expressed as:

$$\frac{\partial(\mathrm{P}(\mathrm{x} \,/\, 0))}{\partial(t)} = p(0) \cdot 0.5 \cdot \left(-\frac{1}{\sqrt{\pi}} \mathrm{e}^{-\left(\frac{\mathrm{t}-\mu(0)}{\sqrt{2}\cdot\sigma(0)}\right)^2} \right) \cdot \frac{1}{\sqrt{2}\cdot\sigma(0)} = -\frac{p(0)}{2\cdot\sqrt{2\pi}\cdot\sigma(0)} \mathrm{e}^{-\left(\frac{\mathrm{t}-\mu(0)}{\sqrt{2}\cdot\sigma(0)}\right)^2}$$

and $\frac{\partial(P(0/x))}{\partial(t)}$ as:

$$\frac{\partial(P(0 \,/\, x))}{\partial(t)} = \sum_{\mathrm{i}=1}^{\mathrm{M}-1} \frac{p(i)}{2\cdot\sqrt{2\pi}\cdot\sigma(i)} \cdot \mathrm{e}^{-\left(\frac{\mu(i)-\mathrm{t}}{\sqrt{2}\cdot\sigma(i)}\right)^2},$$

where M is the number of levels for every discrete case and $\mu(i)$ and $\sigma(i)$ are the mean level value of the signal and its respective total noise parameter ($\sigma_{total\ noise}$), expressed as a Gaussian standard deviation.

Based on the calculation method that is followed, setting the respective derivative to zero, allows for the calculation of the optimum threshold (f(t) = 0):

$$f(t) = -\frac{p(0)}{2\cdot\sqrt{2\pi}\cdot\sigma(0)} e^{-\left(\frac{t-\mu(0)}{\sqrt{2}\cdot\sigma(0)}\right)^2} + \sum_{i=1}^{M-1} \frac{p(i)}{2\cdot\sqrt{2\pi}\cdot\sigma(i)} \cdot e^{-\left(\frac{\mu(i)-t}{\sqrt{2}\cdot\sigma(i)}\right)^2} = 0$$

By applying the natural logarithm and rearranging the equation, it then can be expressed in a standard quadratic form, as follows: $A\cdot t^2 + B\cdot t + C = 0$, where the coefficients are defined as:

$$A = \frac{1}{2\cdot\sigma(0)^2} - \sum_{i=1}^{M-1} \frac{p(i)}{2\cdot\sigma(i)^2}$$

$$B = -\frac{\mu(0)}{\sigma(0)^2} + \sum_{i=1}^{M-1} \frac{\mu(i)}{\sigma(i)^2}$$

$$C = \frac{\mu(0)^2}{2\cdot\sigma(0)^2} - \sum_{i=1}^{M-1} \frac{\mu(i)^2}{2\cdot\sigma(i)^2} - \ln\left(\frac{p(0)}{\sum_{i=1}^{M-1} p(i)} \frac{\prod_{i=1}^{M-1}\sigma(i)}{\sigma(0)}\right)$$

Solving this equation allows for yielding the optimum threshold, for the calculation of the MER.

Finally, based on all the previously presented methods the Supplementary equation (5) for the calculation of the MER is expressed as:

$$MER = P(x\,/\,0) + P(0\,/\,x) = p(0)\cdot 0.5\cdot \mathrm{erfc}\left(\frac{t-\mu(0)}{\sqrt{2}\cdot\sigma(0)}\right) + \sum_{i=1}^{M-1} p(i)\cdot 0.5\cdot \mathrm{erfc}\left(\frac{\mu(i)-t}{\sqrt{2}\cdot\sigma(i)}\right)$$

Conversely, the calculation of the total SER of the signal is based on the following equation:

$$SER_{total} = \sum_{i=0}^{M-1} p(i)\cdot SER(i) \tag{7}$$

where $SER(i)$ expresses the symbol error rate of each output word comparison (signal level).

The calculation of the overall SER incorporates the respective MER of each case, assuming that SER(0) = MER and is calculated with the method mentioned above. For all the signal levels that range between the levels 1 to M-2 [1, M-2], the calculation of the respective SER(i) is based on the following equation:

$$SER(i) = p(i)\cdot 0.5\cdot \mathrm{erfc}\left(\frac{\mu(i)-t(i-1)}{\sqrt{2}\cdot\sigma(i)}\right) + p(i)\cdot 0.5\cdot \mathrm{erfc}\left(\frac{t(i)-\mu(i)}{\sqrt{2}\cdot\sigma(i)}\right) + \sum_{j=i+1}^{M-1} p(j)\cdot 0.5\cdot \mathrm{erfc}\left(\frac{\mu(j)-t(j)}{\sqrt{2}\cdot\sigma(j)}\right) + \sum_{k=i-1}^{0} p(k)\cdot 0.5\cdot \mathrm{erfc}\left(\frac{t(k-1)-\mu(k)}{\sqrt{2}\cdot\sigma(k)}\right) \tag{8}$$,

while in the case of the last signal level (i.e. M-1) the calculation of the SER(M-1) is expressed as:

$$SER(M-1) = p(M-1)\cdot 0.5\cdot \mathrm{erfc}\left(\frac{\mu(M-1)-t(M-2)}{\sqrt{2}\cdot\sigma(M-1)}\right) + \sum_{j=M-2}^{0} p(j)\cdot 0.5\cdot \mathrm{erfc}\left(\frac{t(j-1)-\mu(j)}{\sqrt{2}\cdot\sigma(j)}\right) \tag{9}$$,

The optimal thresholds are also calculated in order to acquire the most accurate SER measurements. Towards determining the optimal thresholds, the numerical iterative approach of the Newton-Raphson method is exploited[6]. Specifically, this method is employed for the approximation of the root of the threshold function that equals to 0, by using the iterative formula:

$$X_{n+1} = X_n - \frac{f(x)}{f'(x_n)} \tag{10}$$,

where X_n is the current approximation, while X_{n+1} is the new approximation value of the respective function. In the current implementation, after approximately 100 iterations, the optimal thresholds corresponding to the minimum SER values are selected. By applying the respective threshold values in the Supplementary equation (7), the total SER of the system can be calculated.

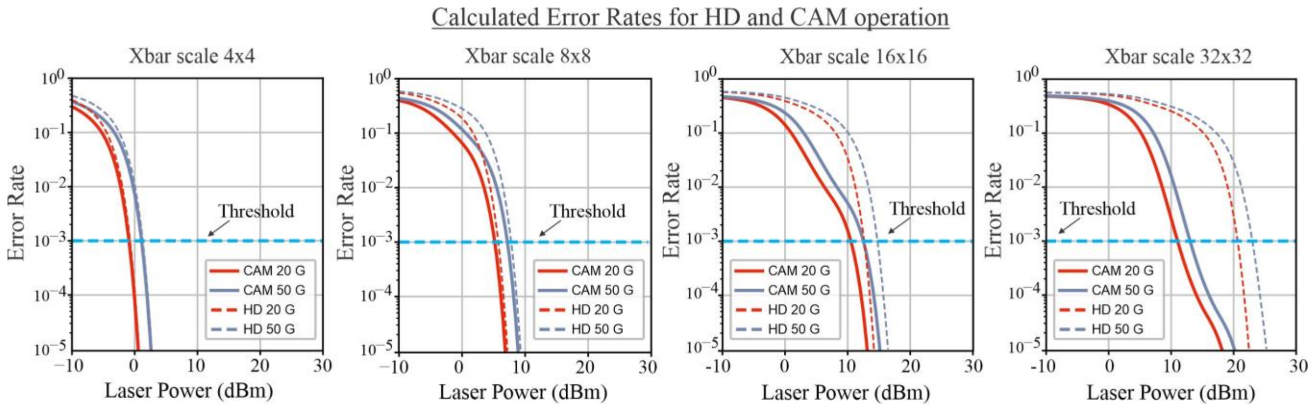

Supplementary Fig. 5. Calculated HD and CAM error rate values for crossbar scales of 4x4, 8x8, 16x16 and 32x32 for the data rates of 20 to 50 GHz. The diagrams indicate the error rate response across a laser power range of 40 dB (from -10 to 30 dB), indicating the required laser power in order to achieve error rates of 10^{-3} (threshold).

Supplementary Figure 5 demonstrates the diagrams of the acquired error rate values of the analysis, compared to the required laser input power of the system for the Xbar scale cases of 4x4, 8x8, 16x16 and 32x32 when targeting a single λ architecture, for performing the respective HD and CAM operations, at both 20 and 50 GHz. The diagrams indicate the need for higher power as the scale of the Xbar implementation and the operational data rates increase, in order to achieve error rate values of 10^{-3}, comparing the capabilities of the current layouts with state-of-the-art electronic demonstrated devices. This mainly occurs due to the increase of the overall losses of the architecture for the larger Xbar scales, as well as the enhanced noise contribution of the higher rate operations. In the crossbar scale cases of 16x16 and 32x32, a relatively different response characterises the error rate curves that are assigned to the respective CAM operations of the two different operational rates under study. Specifically, as the laser power increases, the noise percentage characterizing the error rate response of each signal level decreases more slowly from the highest to the lowest power signal levels. Considering also that each signal level is associated with unequal probabilities of occurrence (follows a binomial distribution) at the output of the HD comparison architecture, this effect is reflected in the corresponding graphs. The current analysis showcases that the single-wavelength layout can efficiently perform comparison operations up to a 32x32 Xbar scale, especially for the cases of the CAM-only matching functionalities at high operational data rates, since the required laser powers for these operations at both data rates, do not exceed the values of 13 dBm for 10^{-3} error rate values.

The error rate analysis of the WDM HD and CAM operator follows the same principles, regarding the contributed noise sources of the architecture and the calculation of the respective MER and SER measurements. Additionally, the experimental MERs and SERs, provided in the main manuscript, are calculated following the same methodology mentioned above, with the difference being that the signal levels and the respective noise parameters of every experimentally measured case, are extracted from the acquired experimental signals.

4. Power consumption analysis of single wavelength and WDM-enabled HD processors

The overall power requirements of the architectures have been evaluated for all the mentioned cases. The power consumption parameters that are included in the current implementation are the respective required laser sources (TLSs), the total power consumption of the Xbar layout, the respective transimpedance amplifiers (TIAs) as receiver circuitry, and the decision units (DDs) responsible for distinguishing the logical comparison operations, as shown in Supplementary Figure 3.

Starting with the laser consumption, different laser powers are considered for the single λ and the WDM cases, with the values being selected based on the threshold required powers (P_{laser}), for 10^{-3} operations. The laser consumption is then calculated using the following equation:

$$\text{Consumption}_{laser}\ (\text{mW}) = \text{P}_{laser}\ (\text{mW}) \ /\ \text{wall-plug efficiency} \quad (11),$$

with a laser wall-plug efficiency of 0.1 (10 % output laser power compared to the electrical power that the device receives in order to operate).

The consumption of the crossbar-based architectures includes the switching energy of statically biased EAMs, the power consumption of the phase shifter (PS) modules, as well as the respective digital to analog (DAC) modules that are required to drive the respective components. The consumption of the thermos-optic PS components has been assigned to constant values of 4 mW[7], while the consumption of the static DACs to 1.74 nW[8].

Regarding the EAMs, we consider that the biasing voltage for every component will be -1.5V (V_{bias}), given that half of the EAMs will be biased at -3V (i.e. the "OFF" state) and the others will be biased at 0V (i.e. the "ON" state) at a specific time. Moreover, we consider that the responsivity (R) of the EAMs is 0.8 A/W, and the inserted input power values (P_{input}) are calculated based on the total path losses of the layout, taking into consideration all the splitting factors of the Xbar. Hence, the total power consumption for each EAM has been calculated based on the equation:

$$\text{Consumption}_{EAM}\ (\text{mW}) = \frac{1}{2} \cdot \text{P}_{input} \cdot \text{R} \cdot \text{V}_{bias} \quad (12)$$

Additional information can be found in[9].

Basen on the calculation of every Xbar component consumption, the total consumption of every different Xbar-scale layout can be computed as follows:

$$\text{Consumption}_{crossbar}\ (\text{mW}) = \text{N}^2 \cdot (\text{Consumption}_{EAM} + \text{Consumption}_{ps}) + 2 \cdot \text{N}^2 \cdot \text{Consumption}_{DAC} \quad (13),$$

where N^2 is the total number of EAM and PS modules of every different crossbar case, along with the $2 \cdot N^2$ static DACs.

Following the crossbar consumption, the energy requirements of the TIA components are also taken into account. Specifically, a constant consumption is considered for the TIAs, with a consumption of 3.5 mW[10] for the data rate of 20 GHz, and a consumption of 59 mW for the data rate of 50 GHz[11].

Moreover, the consumption of the comparator decision units (DDs) is also considered in the total consumption of the architecture. The comparator unit is based on a strong-arm latch topology[12], with the following equation expressing its consumption profile:

$$\text{Consumption}_{DD}\ (\text{mW}) = \text{B}/2 \cdot (2 \cdot \text{C}_{P,Q} + \text{C}_{X,Y}) \cdot \text{V}_{DD}^2 \quad (14),$$

where B is the operational data rate, $C_{P,Q}$ and $C_{X,Y}$ being the comparator capacitors with values of 200F each and V_{DD} the supply voltage of the device with a value of 0.9V. Based on the Supplementary equation (14) the consumption increases as the operational data rate increases as well.

Following the Supplementary equations (11), (12), (13) and (14), the total power consumption is calculated as:

$$\text{Consumption}_{Total}\ (\text{mW}) = \text{Consumption}_{laser}\ (\text{mW}) + \text{Consumption}_{crossbar}\ (\text{mW}) + \text{M} \cdot \text{Consumption}_{TIA}\ (\text{mW}) + \text{M} \cdot \text{Consumption}_{DD}\ (\text{mW}) \quad (15),$$

with M being the number of TIAs and DDs at every output column of the crossbar.

After calculating the consumption for all the different crossbar scales and data rates, the energy efficiency (pJ/bit) is also calculated for all the respective cases. The efficiency values are calculated based on the total power consumption of the architecture and the respective data rate value (B), taking also into account the total number of output compared words at every specific scale architecture, as well as the number of symbols (symbol-length) of every output word. Particularly, the energy efficiency is calculated as:

$$\text{Energy Efficiency (pJ/bit)} = \frac{\text{ConsumptionTotal (W)}}{\text{K} \cdot \text{L} \cdot \text{B (bits/second)}} \cdot 10^{12} \quad (16),$$

where L is the total number of output words and K is the length of every specific word.

The WDM-enabled architecture can introduce a capacity increase of the comparison layout, since the utilization of additional wavelengths can offer additional comparison operations at a given Xbar scale. Specifically, an additional analysis is performed, considering the use of four distinct wavelength cases (λ=4). Based on that, the single-wavelength scale layouts can be reduced according to the capacity wavelength factor of four. Following this principle the Xbar scale of the single λ cases of 4x4 to 128x128 can be adjusted to scales of 4x1 to 128x32, for the proposed WDM-enhanced architectures. This approach allows for minimization in the overall scale of the layout, allowing for a reduced number of output columns, thus reducing the splitting and combination required components achieving the same number of comparison operations. Based on that, due to the reduced splitting stages the required laser power launched into the crossbar array will be minimized, since less power will be needed in order to propagate the same power value at every column of the array. On top of that the component losses of the splitter/combiner stages will be also reduced, with the only additional loss parameter being the Mux and Demux stages that the different wavelengths will propagate through (3 stages for every column). Considering state-of-the-art low-loss multiplexer and demultiplexer components that can be utilized in the WDM-architectures, a minimal losses of 0.2 dB per Mux/De-Mux module[14], can be estimated.

This leads to significantly reduced overall insertion losses for the WDM implemented schemes (IL_{WDM}) compared to the total losses of the conventional Xbar layouts ($IL_{single\text{-}wavelength}$), with the loss reduction being correlated to the number of allocated wavelengths that are used for the parallel comparisons and the reduced columns of every layout. Such an approach significantly reduces the overall insertion losses of the architecture, especially when scaling to 32 and 64-bit long comparison schemes.

The method of calculating the total losses of the conventional architecture ($IL_{single\text{-}wavelength}$) are thoroughly explained in[13], while the loss reduction parameters of the WDM layouts are provided below for every respective scale case of [4-128]:

- Xbar scale 4x1 → 5.66 dB loss reduction
- Xbar scale 8x2 → 5.86 dB loss reduction
- Xbar scale 16x4 → 6.4 dB loss reduction
- Xbar scale 32x8 → 7.6 dB loss reduction
- Xbar scale 64x16 → 10.9 dB loss reduction
- Xbar scale 128x32 → 19.8 dB loss reduction

Based on the loss reduction parameters introduced by the WDM layouts, the required laser powers for the newly proposed schemes can be calculated. The laser power reduction is performed according to the loss reduction factor offered by every WDM-Xbar based layout adjusted to a specific power number ($P_{reduction}$). Following the Supplementary equation (11) the calculation of the laser consumption in the WDM cases is configured as:

$$\text{Consumption}_{WDM\ laser}\ (\text{mW}) = [(P_{laser}\ (\text{mW}) - P_{reduction}\ (\text{mW}))\ /\ \text{wall-plug efficiency}] \cdot \lambda \quad (17),$$

where in the specific case the total laser consumption of the WDM layout will be equal to the consumption of all the utilized laser sources (λ=4).

In Supplementary Figure 6 the required laser powers when utilizing the single or 4-λ layouts for the HD and CAM operations are presented, along with the respective energy efficiency values across the different Xbar scales, for both 20 and 50 Gb/s. The diagrams indicate that the use of multiple wavelengths (four in the specific case), instead of a single wavelength can significantly reduce the overall losses as well as the consumption requirements of the layouts, especially when the Xbar scales up to the 64 and 128 implementations. As also shown in Supplementary Figure 6 (b) and (d) energy efficiencies of 400 fJ/bit and 200 fJ/bit can be achieved respectively, for the WDM CAM operations, while for the WDM HD operations the energy efficiency values remain below the 800 fJ/bit, thus highlighting the efficiency of the WDM-

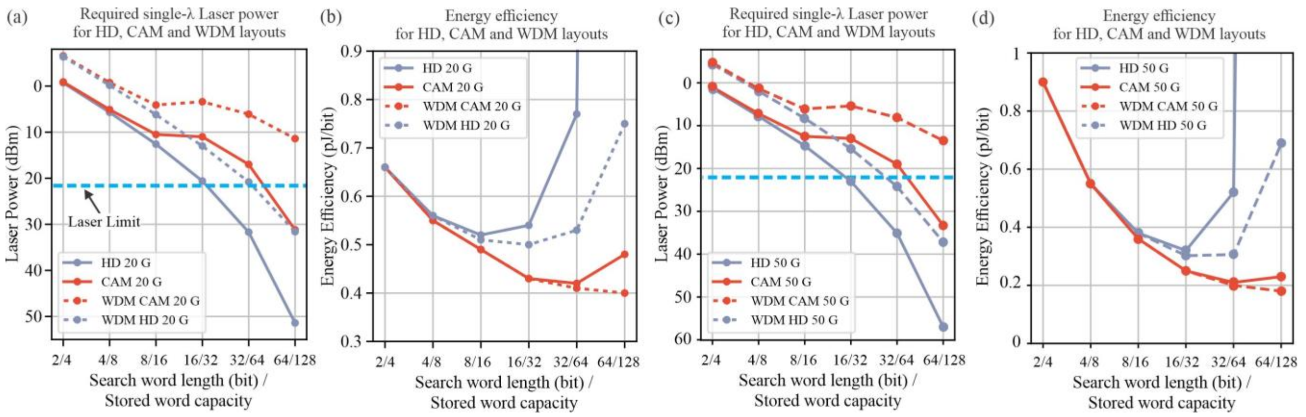

Supplementary Fig. 6. (a) Required laser power for single-λ and WDM HD,CAM layouts for search word length of [2-64] and capacity of [4-128], for the 20 Gb/s operations, (b) Energy efficiency (pJ/bit) for the respective layouts at 20 Gb/s. (c) and (d) Respective cases for the 50 Gb/s operations.

layouts even for the largest Xbar scale. Furthermore, the higher scale word-length CAM and HD functionalities can realistically be performed, especially in the 32-bit comparison case, using state-of-the-art laser technologies (referenced in the main manuscript). In that case, the laser consumption of the WDM cases can still be compensated compared to the single λ cases, were the laser becomes the dominant consumption factor of the architecture, with this effect being presented in the energy efficiency diagrams.

5. <u>Crosstalk and Extinction Ratio analysis</u>

<u>Waveguide Crossings-induced crosstalk:</u> This section investigates the impact of crossing-induced crosstalk on the performance of the HD processor. Considering the deployment of waveguide crossings presented in the work of reference[15], that demonstrated -55 dB of crosstalk, we performed a simulation analysis, based on this specific value. For the theoretical model we used a Monte Carlo analysis to evaluate the impact of crosstalk and scaling on the performance of our architecture. The study involves varying the size of a square crossbar array, across values of N = [4, 8, 16, 32, 64, 128], while maintaining a fixed crosstalk level of -55 dB. For each crossbar scale, the simulation conducts 100 trials to compute the dot product between the search and target vectors, both initialized with discrete binary values. The dot products are calculated under two conditions: (i) an ideal scenario and (ii) a noisy scenario where crosstalk-induced interference is introduced to the layout. In the case where noise is applied, the dot products are derived by applying a crosstalk factor, corresponding to the -55 dB and additionally, random phase shifts ranging from [0-2pi] are considered for the incoming search vector's signals. Following this, the average error between the ideal and noisy dot products is then calculated. Supp. Fig. 7 illustrates the simulation results showing the average dot product error, induced by the waveguide crosstalk, for different crossbar scales. As shown, the performance degradation due to crosstalk remains negligible even for crossbar scales as high as 128x128, reaching only ~8×10^{-3} %, showcasing that this specific crosstalk parameter does not limit the scalability of the architecture.

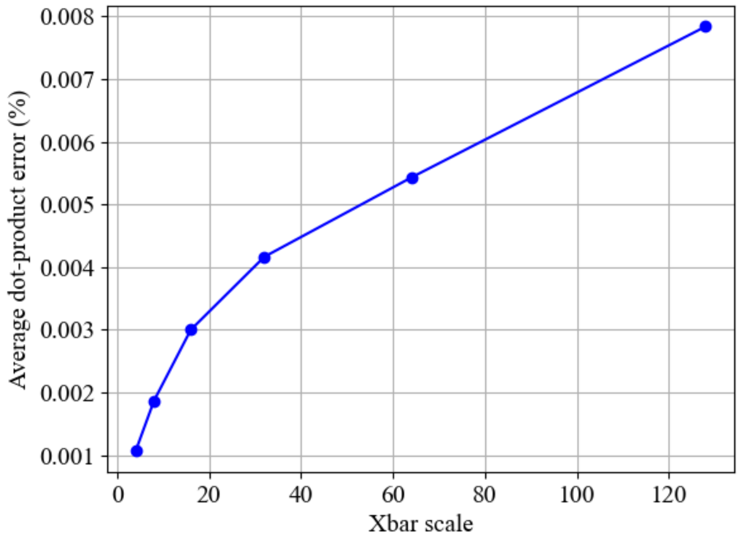

Supplementary Fig. 7: Average dot product error, induced by the crosstalk of waveguide crossings for various Xbar scales.

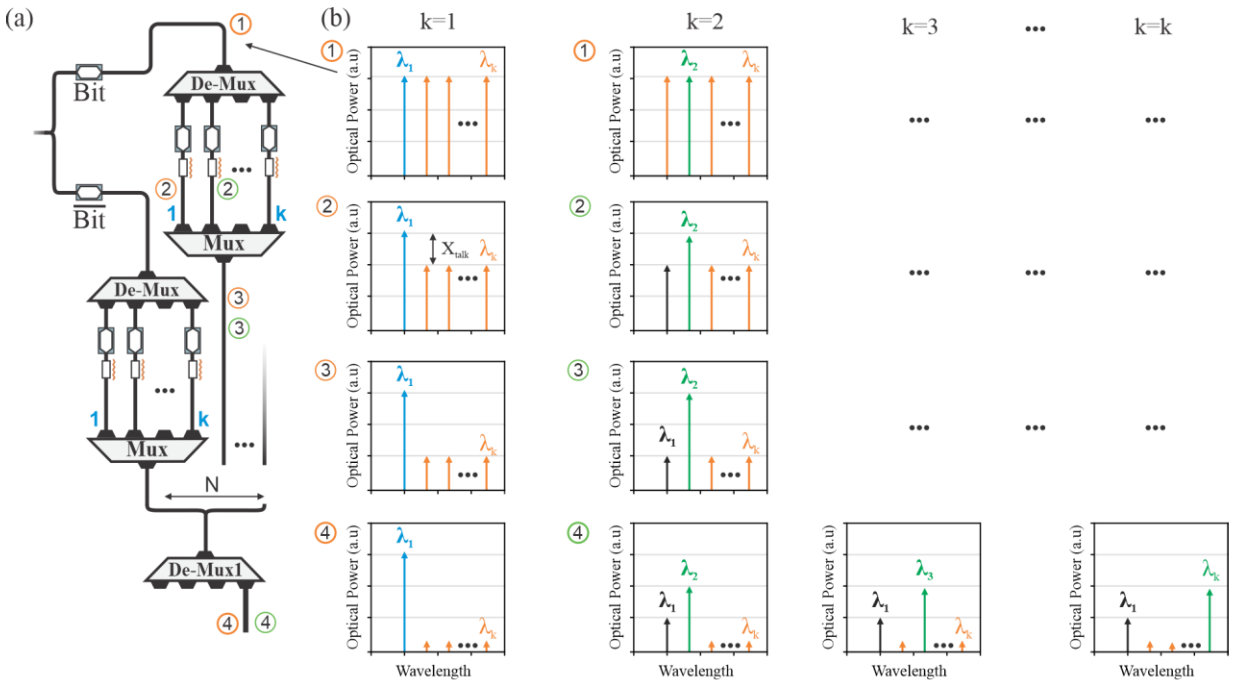

Supplementary Fig. 8: (a) Equivalent circuit layout of a WMD-Xbar with k wavelength channels and (b) Schematic illustration of crosstalk aggressors for single column operation.

(De)Multiplexers-induced crosstalk:

This section investigates the mux-induced optical crosstalk that might affect the performance of the WDM crossbar layout, particularly as the number of wavelength channels increases and/or the channel isolation performance of the underlying photonic components decreases. In order to quantify the effects of the optical crosstalk in a k number of wavelength channels WDM-crossbar, we begin by depicting in Supp. Fig. 8 (a) the equivalent circuit layout of a single column of the WDM layout. We also assume a crosstalk value of A dB and symmetric channel operation. Supp. Fig. 8 (b) illustrates the optical power evolution of the multiwavelength signals traversing the crossbar layout for each of the k wavelength channels. By denoting λ_1 as the signal of interest and identifying all the other wavelength channels as crosstalk inducing aggressors, we conclude to the following:

- Channel 1 crosstalk power:
  - $P_{xtalk1} = (k - 1) * (P_{input} * 3 * MUX_{xtalk})$ (18)

where P_{input} is the optical power for each wavelength channel at the input of the first De-MUX, while MUX_{xtalk} is the isolation of each of the WDM components in linear form.

- Channel 2-k crosstalk power:

$$P_{xtalk2} = (P_{input} * 2 * MUX_{xtalk}) + (P_{input} * MUX_{xtalk}) \quad (19)$$

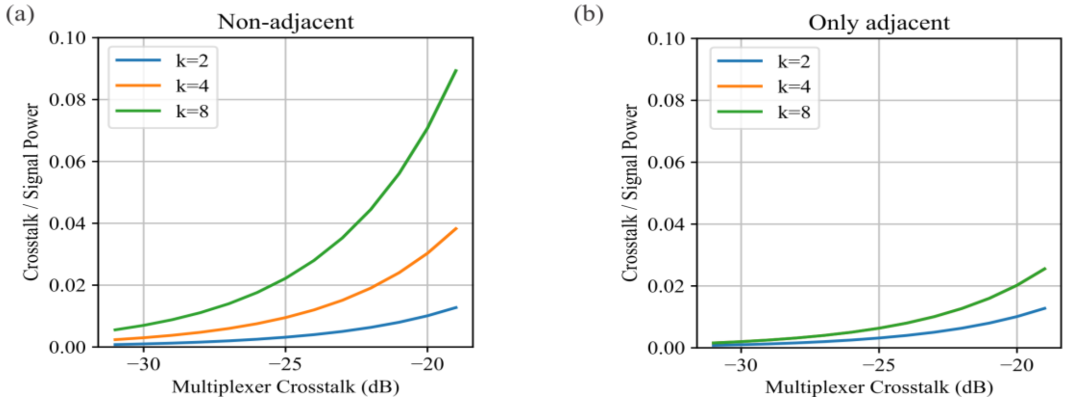

Supplementary Fig. 9: (De)Mux-induced Crosstalk to signal power (a) Assuming symmetric crosstalk performance across or multiplexer channels and (b) Assuming only adjacent channel crosstalk.

$$+ (k-2) * (P_{input} * 3 * MUX_{xtalk}),$$

with the first component being the intra-band crosstalk at λ_1, the second component originating from $\lambda_{2(\kappa=2)}$ and the (k-2) components corresponding to the crosstalk of all channels to the signal bearing signal.

Adding up these contributions we conclude to:

$$P_{xtalk} = P_{xtalk1} + \mathbf{(k-1)} * P_{xtalk2} \quad (20)$$

$$(k-1) * (P_{input} - 3 * MUX_{xtalk}) + k * \{(P_{input} - 2 * MUX_{xtalk}) + (P_{input} - MUX_{xtalk}) + (k-2) * (P_{input} - 3 * MUX_{xtalk})\}.$$

It should be noted that typical SiPho MUX/DEMUX designs have significant crosstalk only on the case of the adjacent channels, and as such the **(k-1)** factor on the P_{xtalk2} can be reduced to 2.

Supp. Fig. 9 (a) and (b) illustrate the relationship between crosstalk to signal power for different multiplexer isolation (crosstalk) and wavelength configurations, taking into consideration the cases of adjacent and non-adjacent crosstalk contributions. Specifically, Supp. Fig. 9 (a) and (b) illustrate the scenarios where crosstalk is symmetric across all channels and when only adjacent channels contribute to crosstalk power. As can be observed for the case of the adjacent channel crosstalk, a typical performance of -27 dB of crosstalk[16] results in a negligible crosstalk to signal power of 0.004 or 0.4 % and as such we conclude that it would not have any significant effect on the error rates performance.

Extinction Ratio analysis: One of the main sources of error-rate degradation of our processor is the limited ER of the search-vector-encoding EAMs. These EAMs operate at GHz regime exhibiting an ER of ~3 dB, while the EAMs responsible for CAM table encoding operate in a static dc regime and have a sufficiently high ER of ~8 dB. However, the resulting 3-level signal will be bounded by the maximum and minimum power levels of the search-vector bits, implying that now 3 different power levels have to be accommodated within an ER of 3dB between the maximum and minimum power level. To assess the impact of the search vector's ER on the error rate, we performed a simulation analysis projecting the error rates of a 4x4 Xbar as a function of the received average power for 50 Gb/s HD and CAM operation under different ER conditions. Supp. Figure 10 depicts the simulation results, clearly highlighting that higher ER values reduce the receiver's sensitivity needs, requiring lower power values to achieve the same error rate threshold. This suggests that adopting a different modulator technology for search vector encoding could enhance overall system performance. For example, MRRs typically exhibit ER values of 6 dB 9, which, according to Figure 10, corresponds to a 37% reduction in the receiver's required power to achieve an error rate of 10^{-3}. A further reduction of over 50% can be achieved by employing Mach-Zehnder Modulators (MZMs) for search-vector-encoding, which can yield ER values of ≥10 dB[17]. However, each modulator technology introduces distinct requirements and limitations. For instance, MRRs are sensitive to temperature variations, while MZMs significantly increase the footprint. These trade-offs should be carefully evaluated when designing a fully integrated system in order to apply the proper mitigation strategies and finally ensure optimal performance.

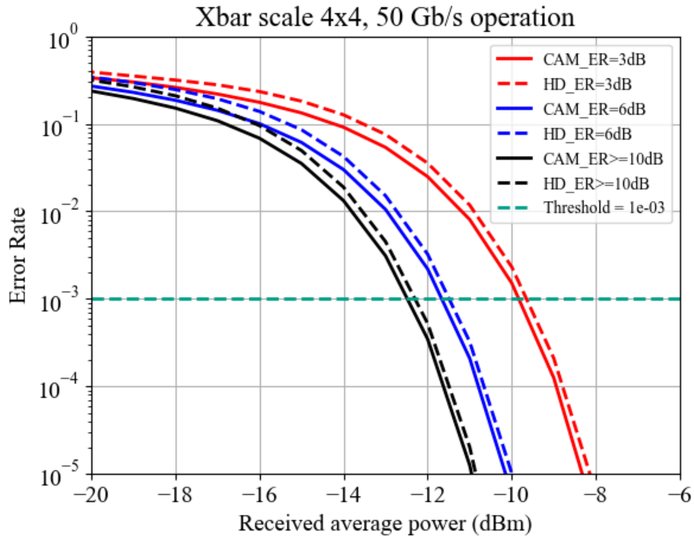

Supplementary Fig. 10: Projected error rates for a 4x4 Xbar implementation at 50 Gb/s CAM and HD operation under different search vector's ER conditions. An ER of 6 dB corresponds to microring modulators, while ER ≥10 dB corresponds to Mach-Zehnder modulators.

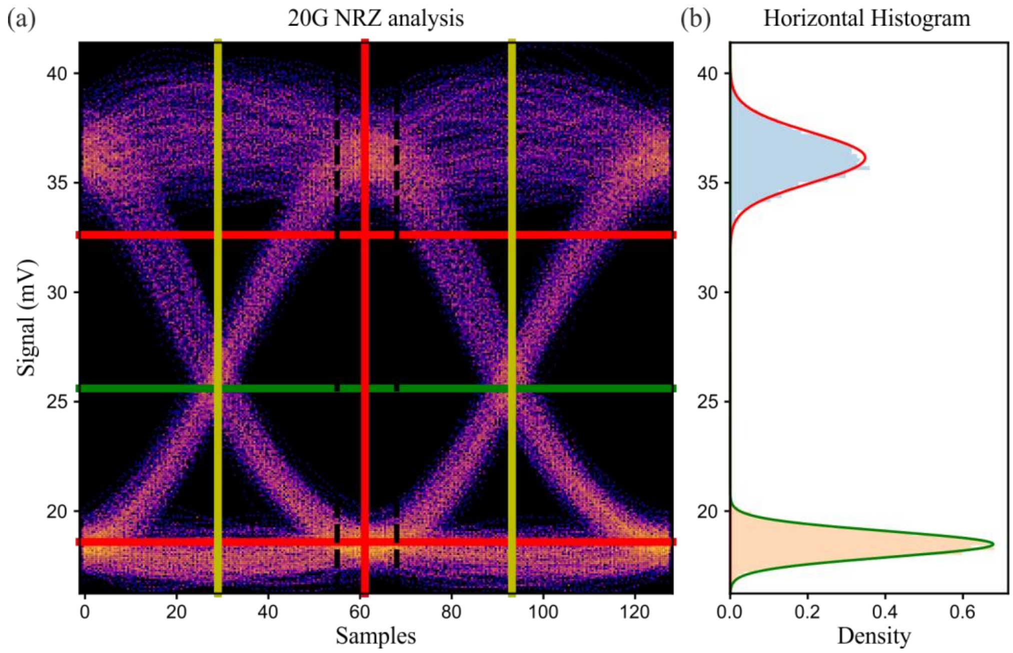

(c)

Parameter	Value
Qfactor	10.183
ER (dB)	2.912
Lvl1 mean	36.146
Lvl0 mean	18.488
Lvl1 std	1.146
Lvl0 std	0.588

Supplementary Figure 11: (a) Optical Eye Diagram of 20 Gbit/s NRZ signal (b) Horizontal histogram in the 10% boundary of the X-axis center (c) Fitted gaussian distributions characteristics for binary zero and one levels.

6. Experimental noise analysis: Impact of different noise sources

Considering the break-down of the noise sources and the relationship with the achieved MER/SER, our approach is two-fold:

- The simulations results presented in the discussion section of the manuscript, are derived from an in-house developed optical simulator, that considers shot, thermal and RIN noise as extensively discussed in Supplementary Section part 3.
- A quantitative analysis of the noise contributions in the experimental results presented in Figure 3 of the main manuscript follows.

Our analysis begins by depicting in Figure 3, the optical eye-diagram analysis of the 20 Gbit/s NRZ signal. Specifically, Supp. Fig. 11 (a) depicts the optical eye diagram, with the yellow, red and green lines corresponding to the crossing points, the 20%, 80% transitions, and the Y axis eye center, respectively. Supp. Fig. 11 (b) illustrates the probability density plots at the 10% percentage boundary of the X-axis eye center for the two binary levels. Fitting a gaussian distribution in each binary symbol class allows the calculation of the mean and standard deviation values of the noise profiles, with the respective results along with other eye-diagram metric illustrated in Supp. Fig. 11 (c). Observation of the experimental data leads to some important conclusions:

- The noise profiles at both binary levels follows a gaussian distribution.

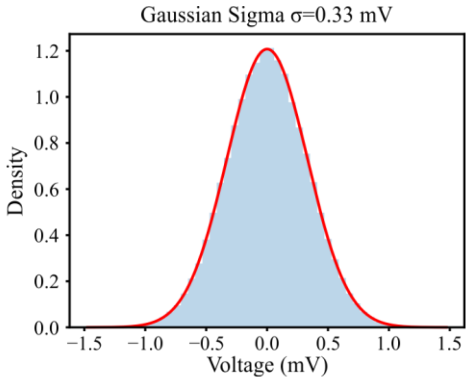

Supplementary Figure 12: Probability density plot of the signal at the digital sampling scope, after a 20 GHz low pass filter.

- As the noise profile of the '1' binary level is significantly higher than the respective '0' level, the expected noise seems to be dominated by a receiver power dependent phenomenon, suggesting that thermal noise is not the highest noise contributor.

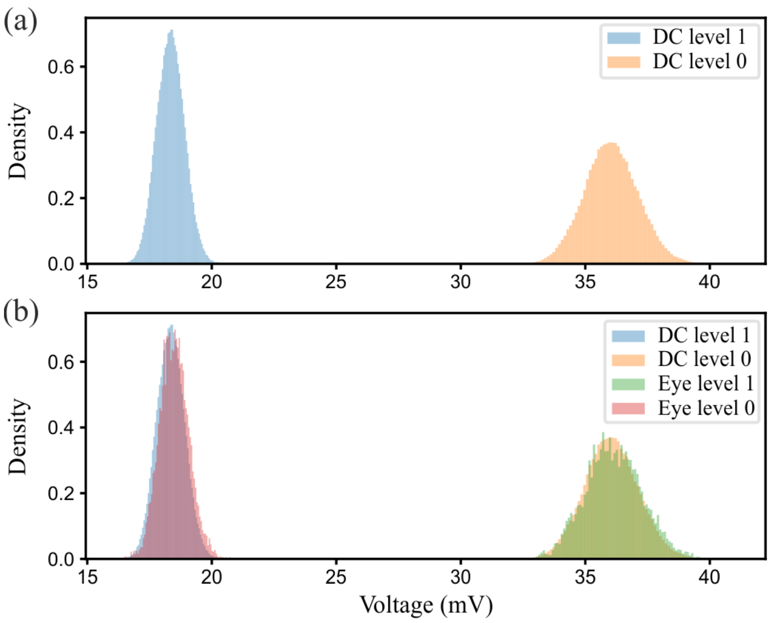

Supplementary Fig. 13 (a) Probability density plot of DC measurement of noise profile at binary level '0' and '1' at 20 Gb/s (b) Overlayed DC and RF noise profiles

To quantify the contribution of the digital sampling scope thermal noise, we plot in Supp. Fig. 12, the probability density plot of a scope measurement, when no input power is injected and a 20 GHz low-pass filter is applied to the sampling scope signal. Fitting a gaussian distribution to the density plot reveals a standard deviation of 0.33 mV, that as expected is significantly lower than the measured 0.58 and 1.1 mV standard deviations at the zero and one binary levels respectively.

Having isolated the thermal noise contribution of the receiver, we experimentally replicated, using DC signals, the opto-electronic component chain employed in our experiments. Specifically, a tunable laser source tuned to the same wavelength as in the experiment, was attenuated to match the power level arising at the chip output (-27 dBm). Following the attenuated signal was injected into a EDFA amplifier, filtered in a 1 nm 3dB bandwidth optical filter and finally injected into a photodiode and sampled in the digital oscilloscope. The injected power at the photodiode was tuned based on the mean values of the binary zero and one levels of the previously acquired eye diagram (i.e. 18.4 and 36.1 mV, respectively). Supp. Fig. 13 (a) illustrates the experimental derived probability density plot for the DC measurements for both binary levels 0 and 1. The respective plots are overlayed in Fig. Supp. Fig. 13 (b) with the optical eye diagram experimental derived probability density plots, revealing perfect matching. As such, the increased noise profiles at the optical eye diagram

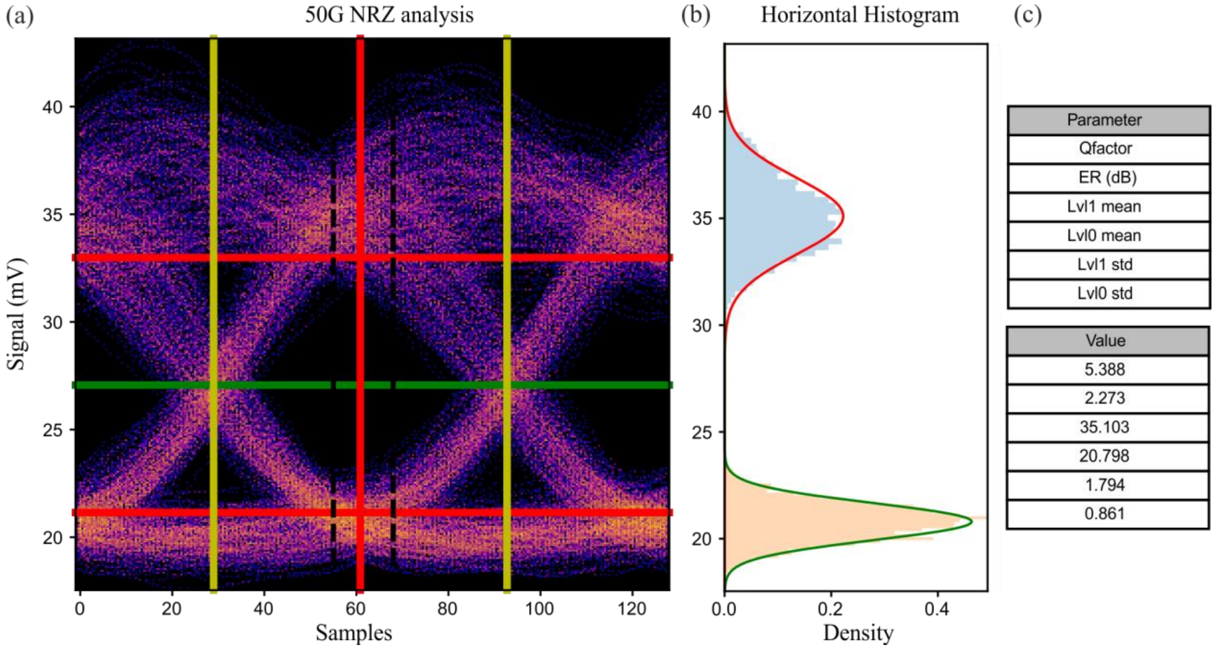

Supplementary Fig. 14: (a) Optical Eye Diagram of 50 Gbit/s NRZ signal (b) Horizontal histogram in the 10% boundary of the X-axis center (c) Fitted gaussian distributions characteristics for binary zero and one levels.

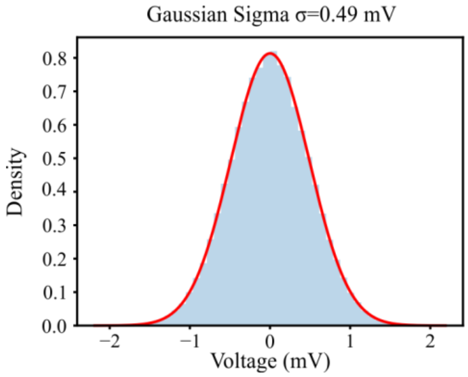

Supplementary Fig. 15: Probability density plot of the signal at the digital sampling scope, after a 50 GHz low pass filter.

measurements, as compared to the scope's thermal noise, is attributed to the EDFA originating noise, with the largest contribution attributed to the beating of the EDFA's ASE with the signal power, resulting in a receiver power dependent noise profile[18]. The previous analysis is repeated for 50 Gb/s NRZ signals, with Supp. Figs. 14, 15 and 16 illustrating the thermal noise after a 50 GHz low pass filter, the optical eye diagram and respective metrics and the overlayed DC and RF density plots, respectively. It is evident that the noise profiles at 50 Gb/s operations is increased due to higher thermal and signal-to-ASE beating noise at 50 GHz. However, the overlayed plots showcase almost perfect matching, distorted only slightly at the '1' binary level, due to the eye skew originating from the limited frequency response. This analysis leads to the following conclusions, considering the eye closure between 20 and 50 Gb/s operations.

- While the electro-optic frequency response is indeed limited, its effect on the eye opening is significantly minimized through a pre-emphasis procedure. However, the pre-emphasis procedure results in a reduction in the achieved ER, from 2.9 dB to 2.1 dB, when transitioning from 20 to 50 Gb/s operation.
- The increased noise profile at higher operating rates and as such operating bandwidths, is also a significant contributor in the reduction of the achieved eye opening.

Quantifying these distortions (ER and increased noise profile) in terms of theoretically achieved Bit Error Rate, can be performed through the use of optical eye Q-factor:

$$Q = \frac{P_1 - P_0}{\sigma_1 + \sigma_0} = \frac{P_1 - P_1/ER}{\sigma_1 + \sigma_0} = P_1 * \frac{1 - \frac{1}{ER}}{\sigma_1 + \sigma_0} \qquad (21)$$

With P_1, P_0, σ_1 and σ_0 corresponding to the mean power levels and noise standard deviation of the signal at binary logic levels '1' and '0' respectively.

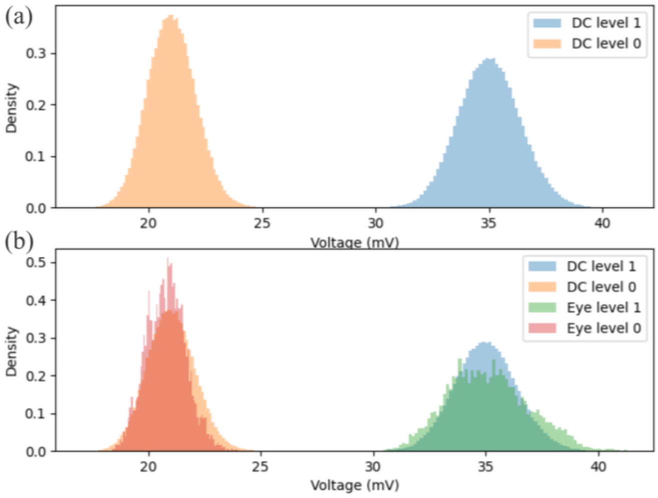

Supplementary Fig. 16 (a) Probability density plot of DC measurement of noise profile at binary level '0' and '1' at 50 Gb/s, (b) Overlayed DC and RF noise profiles.

7. State-of-the-art electronic CAM demonstrations

Ternary features used in vector comparisons, similarity matching and look-up tables require developing hardware TCAMs, that comprise two storage cells, one for the actual data-bit and one for the ternary state. While TCAMs offer simplicity and high performance, they come at the cost of increased power consumption and complex interconnect circuitry. This circuitry broadcasts input data across all TCAM match lines to enable fast parallel comparisons, with the final result collected at the TCAM table output as a single Matchline signal. The first practical integrated electronic CAM was introduced by Koo in 1970[19], yet emerged in the memory market in mid-90s and flourished after 2000, owing to the established Von-Neumann architectures, favouring enhanced search-performance and memory-intensive operations when

compared against conventional electronic RAMs. Since then, TCAMs followed a rapid progress as summarized in the Table 1 that benchmarks various electronics TCAMs optimized for high-performance at various CMOS nodes from 250 nm down to even 2nm.

- Early TCAMs targeted combining the performance of NOR gates with the power efficiency of NAND gates by activating only a few MLs using NAND cells and NOR cells for the rest, as e.g. the device built on 250 nm CMOS node at 260 MHz[20]. Similarly, a TCAM at a 180 nm CMOS node achieved a maximum frequency of 210 MHz[21], while devices on 130 nm CMOS exhibited 200 MHz[22], with area and energy consumptions quickly reaching values of 6.73 µm^2 and 5.6 fJ/bit respectively
- When using CMOS lines of 65 nm, electronic TCAMs achieved to deliver frequencies up to 500 MHz, at an energy efficiency value of 0.2 fJ/bit and footprint of 3.98 µm^2 per cell in 2015 (not simultaneously at the same device) [23-25].
- Although TCAM developed at CMOS nodes between 32nm and 12nm achieved frequencies in the order of 1GHz to 1.5 GHz[26-31], the energy efficiency values were still not improved (values >0.2 fJ/bit are reported), resulting only in 4x increased footprint efficiency of 0.945 µm2 per cell.
- More recently, shifting to the most advanced CMOS nodes of lower than 7 nm[32-35], energy efficiency values were not significantly improved, revealing a plateau of around 0.12 fJ/bit, bounded by the nature of the underlying electrical interconnect.

All this analysis declares that electronic TCAM have energy efficiencies that can reach even sub-fJ/bit values, but they are struggling to advance their data-rate performance. Even with the most advanced electronic nano-sheet layers of NAND-

Supplementary Table 2: State-of-the-art electronic TCAMs

	Ref.	CMOS Techn. (nm)	Area/cell (µm^2)	Energy/search/bit (fJ)	Freq. (GHz)	Array size
Electronic TCAM	19	250	316.2	17.2	0.26	128×32
	20	180	53.2	2.82	0.21	128×144
	22	130	6.73	5.6	0.20	9.4 Mb
	23	65	-	-	0.50	-
	24	65	6.53	0.2	0.13	32 Kbit
	25	65	3.98	0.6	0. 50	8 Kbit
	26	32	0.84	0.58	1.00	2048×640
	27	28	0.6	-	0.40	20 Mbit
	28	28	0.304	0.74	0.37	32×64
	29	16	1.8		1.25	10 Kbit
	30	14	2.01	-	1.40	2K×640 bit
	31	12	0.945	-	1.50	10 Kbit
	32	7	0.248	-	1.60	640 Kbits
	33	5	0.287	-	2.00	512×220
	34	3	0.201	0.305	2.20	512×220
	35	2	0.024	0.120	-	128×128
This Work		SiPho EAM	95,000	32	50	4×4

gates at 2nm nodes, operational-speeds seem to be constrained to less than 2.2 GHz, implying that electronic TCAMs are hard-limited within a few GHz and hence struggle to keep pace with the rising line rates of optical systems.

In this context, photonic CAMs have emerged as a promising alternative computing circuitry, as described also in the main manuscript. The proposed single wavelength and WDM 4x4 TCAM prototypes deliver a tremendous 20x speed-up over the electronic performance plateau, while maintaining reasonable footprint requirements and energy efficiency metrics, being mainly limited by the current photonic integration.

References of the Supplementary Materials

1. P. De Heyn, J. De Coster, P. Verheyen, G. Lepage, M. Pantouvaki, P. Absil, W. Bogaerts, J. Van Campenhout and D. Van Thourhout, "Fabrication-Tolerant Four-Channel Wavelength-Division-Multiplexing Filter Based on Collectively Tuned Si Microrings," Journal of Lightwave Technology, vol. 31, pp. 2785-2792, 2013. DOI: 10.1109/JLT.2013.2273391.
2. https://www.svphotonics.com/pub/pub029.pdf
3. https://en.wikipedia.org/wiki/Central_limit_theorem
4. G. Giamougiannis et. al., "A Coherent Photonic Crossbar for Scalable Universal Linear Optics," in JLT, vol. 41, no. 8, pp. 2425-2442, 15 2023
5. https://en.wikipedia.org/wiki/Binomial_distribution
6. https://www.sciencedirect.com/topics/mathematics/newton-raphson-method
7. IMEC'S Silicon Photonics Platform Services, 2022. [Online]. Available: https://www.imec-int.com/sites/default/files/imported/SILICONPHOTONICS-V06.pdf. [Accessed: 19- Feb- 2022].
8. I. B. Sharuddin and L. Lee, "An ultra-low power and area efficient 10 bit digital to analog converter architecture," 2014 IEEE International Conference on Semiconductor Electronics (ICSE2014), Kuala Lumpur, Malaysia, 2014, pp. 305-308, doi: 10.1109/SMELEC.2014.6920858.
9. M. Pantouvaki et al., "Active Components for 50 Gb/s NRZ-OOK Optical Interconnects in a Silicon Photonics Platform," in Journal of Lightwave Technology, vol. 35, no. 4, pp. 631-638, 15 Feb.15, 2017.
10. S. Saeedi, S. Menezo, G. Pares and A. Emami, "A 25 Gb/s 3D-Integrated CMOS/Silicon-Photonic Receiver for Low-Power High-Sensitivity Optical Communication," in *Journal of Lightwave Technology*, vol. 34, no. 12, pp. 2924-2933, 15 June15, 2016, doi: 10.1109/JLT.2015.2494060.
11. L. Wang, X. Luo, D. Xu, Z. Qiu, Y. Yan and Q. Pan, "A 160-Gb/s 0.37-pJ/bit PAM4 Optical Receiver in 28-nm CMOS," *2022 IEEE Asia Pacific Conference on Circuits and Systems (APCCAS)*, Shenzhen, China, 2022, pp. 333-336, doi: 10.1109/APCCAS55924.2022.10090288.
12. B. Razavi, "The Strong-ARM Latch [A Circuit for All Seasons]," in *IEEE Solid-State Circuits Magazine*, vol. 7, no. 2, pp. 12-17, Spring 2015, doi: 10.1109/MSSC.2015.2418155.
13. A. Tsakyridis et al., "Universal Linear Optics for Ultra-Fast Neuromorphic Silicon Photonics Towards Fj/MAC and TMAC/sec/mm2 Engines," in IEEE Journal of Selected Topics in Quantum Electronics, vol. 28, no. 6: High Density Integr. Multipurpose Photon. Circ., pp. 1-15, Nov.-Dec. 2022, Art no. 8300815, doi: 10.1109/JSTQE.2022.3219288.
14. L. Chenlei et. al., "Low-loss and low-crosstalk multi-channel mode (de)multiplexer with ultrathin silicon waveguides," Opt. Lett., 2017.
15. Tae Joon Seok, Niels Quack, Sangyoon Han, Richard S. Muller, and Ming C. Wu, "Large-scale broadband digital silicon photonic switches with vertical adiabatic couplers," Optica 3, 64-70 (2016).
16. S. Pathak, P. Dumon, D. Van Thourhout and W. Bogaerts, "Comparison of AWGs and Echelle Gratings for Wavelength Division Multiplexing on Silicon-on-Insulator," in IEEE Photonics Journal, vol. 6, no. 5, pp. 1-9, Oct. 2014.
17. C. Li et al., "A 3D-Integrated 56 Gb/s NRZ/PAM4 Reconfigurable Segmented Mach-Zehnder Modulator-Based Si-Photonics Transmitter," 2018 IEEE BiCMOS and Compound Semiconductor Integrated Circuits and Technology Symposium (BCICTS), San Diego, CA, USA, 2018
18. Agrawal, G. P. Fiber-Optic Communication Systems, 4th edn. 261 (John Wiley & Sons, 2012).
19. J.T. Koo, "Integrated-circuit content-addressable memories," IEEE Journal of Solid-State Circuits, volume: 5, issue: 5, pp.208-218, October 1970
20. BD.Yang,L.S.Kim,"A Low-Power CAM Using Pulsed NAND–NOR Match-Line and Charge-Recycling Search-Line Driver," IEEE J. Of Solid-State Circuits, Vol. 40, is. 8, pp.1736-1744, August 2005
21. B.-D. Yang, et. Al., "A Low Power Content Addressable Memory Using Low Swing Search Lines," IEEE Trans. on Circuits and Systems I: Regular Papers , vol. 58, no. 12, pp. 2849-2858, Dec. 2011
22. G. Kasai et al.,"200 MHz/200 MSPS 3.2 W at 1.5 V Vdd, 9.4 Mbits ternary CAM with new charge injection match detect circuits and bank selection scheme"IEEE C. Int.Circuits Conf.,2003, pp. 387–390
23. T. Nagakartnik and J.R. Choi, "500-MHz high-speed, low-power ternary CAM design using selective match line sense amplifier in 65nm CMOS", 6th Inform. and Commun. Syst., pp. 60-63, Amman, 2015
24. E. Garzon, et. al. "A 128-kbit Approximate Search-Capable Content-Addressable Memory (CAM) With Tunable Hamming Distance," IEEE J. of Solid-State Circuits, article in press

25. K. Lee, et. al. "A 65-nm 0.6-fJ/Bit/Search Ternary Content Addressable Memory Using an Adaptive Match-Line Discharge," IEEE J. Solid-Sate Circuits, vol. 56, no. 8, pp.2574-2584, Aug. 2021
26. I. Arsovski, T. Hebig, D. Dobson, R. Wistort, "A 32 nm 0.58-fJ/Bit/Search 1-GHz Ternary Content Addressable Memory Compiler Using Silicon-Aware Early-Predict Late-Correct Sensing With Embedded Deep-Trench Capacitor Noise Mitigation,"IEEE J.Solid-State Circ,vol.48 (4), 932-939, 2013
27. K. N ii, et. al. "A 28nm 400MHz 4-parallel 1.6Gsearch/s 80Mb ternary CAM," IEEE International Solid-State Circuits Conference Digest of Technical Papers, San Francisco, CA, USA, Feb. 2014
28. S. Jeloka, et. al. "A 28 nm Configurable Memory (TCAM/BCAM/SRAM) Using Push-Rule 6T Bit Cell Enabling Logic-in-Memory," IEEE Journal of Solid-State Circuits (Volume: 51, Issue: 4, pp. 1009-1021, April 2016,
29. Y. Tsukamoto, "1.8 Mbit/mm2 Ternary-CAM macro with 484 ps Search Access Time in 16 nm Fin-FET Bulk CMOS Technology," IEEE Symposium on VLSI Circuits, Kyoto, Japan, June 2015
30. I. Avrovski, et. al. "1.4Gsearch/s 2Mb/mm2 TCAM Using Two-Phase-Precharge ML Sensing and Power-Grid Pre-Conditioning to Reduce Ldi/dt Power-Supply Noise by 50%," IEEE International Solid-State Circuits Conference (ISSCC), San Fransisco, CA, USA, Feb. 2017
31. M. Yabuuchi, et. al. "12-nm Fin-FET 3.0G-search/s 80-bit × 128-entry Dual-port Ternary CAM," IEEE Symposium on VLSI Circuits, Honolulu, HI, USA, June 2018
32. M. Yabuuchi, et. al. "A 7nm Fin-FET 4.04-Mb/mm2 TCAM with Improved Electromigration Reliability Using Far-Side Driving Scheme and Self-Adjust Reference Match-Line Amplifier," IEEE Sympos. VLSI Circuits, Honolulu, HI, USA, June 2020
33. C. Deshpande, et. al. "A 5nm Fin-FET 2G-search/s 512-entry x 220-bit TCAM with Single Cycle Entry Update Capability for Data Center ASICs," IEEE VLSI Symposium, Kyoto, Japan, 13-19 June 2021
34. S. Kumar, et. al., "A 3nm FinFET 2.2Gsearch/s 0.305fJ/b TCAM with Dynamically Gated Search Lines for Data-Center ASICs," IEEE Intern. Solid-State Circuits Conf, San Francisco, CA, USA, Feb. 2025
35. L.-A. Yu, et. al. "Design of the 2-nm Nanosheet NAND-type TCAM with High Speed and Compat Cell-size: 45% Layout-reduction of 3-nm TCAM," IEEE Silicon Nanoelectronics Workshop, Honolulu, HI, USA, June 2024
