# Supplementary material for: Nonlinear optical vector processing using linear silicon photonic circuits for 50 Gb/s memory and string similarity functions
Source: Nat Commun. 2025 Dec 17;16:11416. doi: 10.1038/s41467-025-66286-7 (PMC12749030; doi:10.1038/s41467-025-66286-7)
Supplement: Supplementary file 2 — Supplementary Software and Code [file 41467_2025_66286_MOESM2_ESM.pdf]

Codes and Software instructions for submitted manuscript material

The current document includes the basic software requirements and software installation process, for the software used, as well as the code files and data, for the evaluation of the experimental findings and the additional theoretical diagrams and analysis presented in the manuscript.

Software used for the evaluation of experimental data and additional theoretical analysis

The software program that has been utilized is the coding environmental program of PyCharm and all the files consist of python files (.py) for all the diagrams and analysis included in the main manuscript.

The software version utilized is PyCharm Community Edition and is a free programming environment. The oldest version that we used in our computer systems is PyCharm Community Edition 2023.1.3, so this version and all the newest program versions after this can be used to enable the respective code files provided. The PyCharm software program can be downloaded here: https://www.jetbrains.com/pycharm/download/?section=windows

The python version used in all code implementations is python 3.9. This version and all next versions can be used; however, we recommend the version 3.9 for compatibility issues. The respective libraries used for all code implementations must be installed in the respective computer (matplotlib, numpy, math etc.) and based on the python version a respective interpreter version needs to be configured in the PyCharm IDE. All the respective libraries and packages used are included in the source code files.

Python versions can be downloaded here: https://www.python.org/downloads/

There are not any specific computing requirements for the current codes/software, a normal laptop, or desktop system can be used. All required software can be downloaded from the beforementioned links. The typical installation time for all required software versions and libraries is a couple of minutes, depending on the respective computer used. All code implementations require a couple of minutes to run, depending again on the capabilities of the computer that is used and the adjustment of the code parameters respectively. The produced outputs from the respective codes are the diagrams and measurements that are presented in the manuscript and the provided material.

The respective source code files are the following:

- Binomial_probabilities_calculation.py
- Noise_Scale_and_Symbol_probabilities_Analysis_Calculation.py
- Single_Wavelength_Power_Consumption_Analysis.py
- WDM_Power_Consumption_Analysis.py
- Laser_Powers.py
- Experimental_ Error_Analysis_Calculation.py
- Waveguide_crossings_crosstalk_modelling.py
- WDM_crosstalk_modeling.py

Binomial_probabilities_calculation.py:

This code is used to calculate the Binomial distribution probabilities of occurrence that describe every comparison level output of the crossbar matching operator, for the different crossbar scales that are displayed in the analysis of the manuscript (word length: 1, 2, 4, 8, 16, 32, 64). The Binomial distribution histograms are shown in Figure 4, of the supplementary material and are used in the next code implementation (Noise_Scale_and_Symbol_probabilities_Analysis_Calculation.py) for the extraction of the theoretical analysis and experimental value cases.

Noise_Scale_and_Symbol_probabilities_Analysis_Calculation.py:

The specific code implementation includes the steps for evaluating the performance of our proposed multi-comparison architecture in a theoretical basis, considering different noise characteristics of the current system, that can degrade the overall signal performance. The code formula accurately calculates the measurements of the MER and SER, taking into account the noise sources of the laser (RIN noise), the photodiode (shot noise), and the TIA (thermal noise) modules, and utilizes the Binomial probabilities (calculated in Binomial_probabilities_calculation.py), for every respective output power case of the comparison processor unit. The analysis indicates the laser power that is required to be inserted into the system in order to achieve specific error rate values (detailed analysis of the methods is provided in the Supplementary material) for different crossbar scale and data rate cases. The extracted laser power values are used in order to estimate the achievable crossbar scale dimensionality and calculate the energy efficiency characteristics (Single_Wavelength_Power_Consumption_Analysis.py and WDM_Power_Consumption_Analysis.py), provided in the manuscript.

The extraction of the experimental error rate measurements follows the same calculation method, considering the noise characteristics of the experimentally acquired signals.

The diagrams of the respective analysis are shown in Figure 6 of the main manuscript and Figures 5 and 6 of the Supplementary material.

Additionally, in the current analysis the parameter of the extinction ratio (ER) of the search information EAMs is also taken into consideration, for the extraction of the error rate values. As shown, in Figure 2 of our reply letter, different ER values have been considered (3 dB, 6 dB and >10 dB), showing the respective requirements in laser power.

Single_Wavelength_Power_Consumption_Analysis.py:

This code implementation calculates the energy efficiency characteristics of the proposed single-wavelength architecture for different crossbar scales and data rates. The presented formula considers the consumption of the laser source, the overall consumption of the crossbar comparison bank, the receiver (TIA) consumption, as well as the decision unit (DD) consumption. Then, based on the total consumption for each case, the energy efficiency is measured based on the data rate and the scale of the comparison unit. The findings of this analysis are included in Figure 6 of the main manuscript and Figure 6 of the supplementary material.

WDM_Power_Consumption_Analysis.py:

The current code implementation follows the same methodology as the previous one (Single_Wavelength_Power_Consumption_Analysis.py), calculating the energy efficiency for the proposed WDM layout, for the respective crossbar scales and data rates. The analysis implements the scenario of using 4 different wavelengths and shows the capabilities of scaling the WDM architecture to larger crossbar scales, as it introduces reduced losses and thus reduced required laser power, for every case. The additional results of the analysis are shown in Figure 6 of the main manuscript and Figure 6 of the supplementary material.

Laser_Powers.py:

The specific code uses the laser power values that are utilized in the previous code implementations, in order to plot the required laser power values compared to the different achievable crossbar scales, for different error rates, according to state-of-the-art integrated laser power specifications, for both the single wavelength and the WDM layouts.

Experimental_ Error_Analysis_Calculation.py:

This code is used to evaluate the experimentally acquired signals, for all different experimental cases reported in the manuscript, extracting all the respective error rate and Q factor values, as well as the eye diagrams. The formula uses the experimentally captured data (respective provided csv files) and analyses them. The code uses

the data to extract the eye diagrams for every different signal case and then uses these data to calculate the respective error rate values. Depending on the case of the signal (being a produced 3-level output signal or a 2-level signal), the code calculates the MERs, SERs and Q factors/BERs, respectively. The process of calculating the MER and SER values is the same described in Noise_Scale_and_Symbol_probabilities_Analysis_Calculation.py. The parameters that need to be defined in order to validate the experimental cases are described in the code. Some indicative raw data files are also provided in order to test the functionality of the code (Raw Data files), for some of the presented experimental cases. The produced results are shown in Figures 3 and 5 of the main manuscript.

Waveguide_crossings_crosstalk_modelling.py:

This code implements a crosstalk analysis, in order to evaluate the additional crosstalk that is induced by the waveguide crossings of the specific proposed architecture. The analysis considers Monte Carlo simulations for specific state-of-the-art crosstalk values (referenced in the Supplementary material). The analysis has been performed for different crossbar scales (N = 4, 8, 16, 32, 64, 128), for a fixed crosstalk value. The simulation conducted 100 trials for the reproduction of results, for ideal and noisy cases, where for the noisy cases random phase shift is considered for the state of the search vector signals. The respective crosstalk analysis is shown in Figure 1, of our reply letter.

WDM_crosstalk_modeling.py:

The current code performs an analysis regarding the response of our proposed WDM layout, considering the crosstalk parameter that can be observed in the multi-wavelength implementation. We consider k number of wavelength channels (k = 2, 4, 8) for the WDM layout, with constant crosstalk values. The analysis takes into account the cases where we have a symmetric crosstalk channel response and an adjacent crosstalk channel response. The results of the analysis are shown in Figure 3 and Figure 4, of our reply letter.

Additional documentation and instructions are included in every source code file, for every specific case. We recommend to read the inline comments for better understanding of the codes and reproduction of the results.

The Raw data files must be included into the same folder with the code files, especially Experimental_ Error_Analysis_Calculation.py, and the DSP folder must also be included.
